# Biogenic Synthesis of Photosensitive Magnesium Oxide Nanoparticles Using Citron Waste Peel Extract and Evaluation of Their Antibacterial and Anticarcinogenic Potential

**DOI:** 10.1155/2024/8180102

**Published:** 2024-06-06

**Authors:** Nawal M. Al Musayeib, Musarat Amina, Farah Maqsood, Kholoud A. Bokhary, Nada S. Alrashidi

**Affiliations:** ^1^Department of Pharmacognosy, College of Pharmacy, King Saud University, P.O. Box 22452, Riyadh 11495, Saudi Arabia; ^2^Department of Optometry and Vision Science, College of Applied Medical Science, King Saud University, Riyadh 11451, Saudi Arabia

## Abstract

**Background:**

Magnesium oxide nanoparticles (MgONPs) have been fabricated by several approaches, including green chemistry approach due to diverse application and versatile features.

**Objectives:**

The current study aimed to prepare a convenient, biocompatible, and economically viable MgONPs using waste citron peel extract (CP-MgONPs) to evaluate their biological applications.

**Methods:**

The CP-MgONPs were synthesized by a sustainable approach from extract of waste citron peel both as capping and reducing agents without use of any hazardous material. The physicochemical features of formed CP-MgONPs were determined by sophisticated analytical and microscopic techniques. The biogenic CP-MgONPs were examined for their antibacterial, anticarcinogenic, and photocatalytic attributes.

**Results:**

A prominent absorption peak in the UV-Vis spectra at 284 nm was the distinguishing characteristic of the CP-MgONPs. The scanning electron microscopy (SEM) reveals polyhedral morphology of nanoparticles with slight agglomeration of CP-MgONPs. The CP-MgONPs exerted excellent antibacterial potencies against six bacterial strains. The CP-MgONPs displayed significant susceptibility towards *E. coli* (20.72 ± 0.33 mm) and *S. aureus* (19.52 ± 0.05 mm) with the highest inhibition zones. The anticancer effect of CP-MgONPs was evaluated against HepG2 (IC_50_ : 15.3 *μ*g·mL^−1^) cancer cells and exhibited potential anticancer activity. A prompt inversion of cellular injury manifested as impairment of the integrity of the cell membrane, apoptosis, and oxidative stress was observed in treated cells with CP-MgONPs. The biosynthesized CP-MgONPs also conducted successful photocatalytic potential as much as MgO powder under the UV-light using acid orange 8 (AO-8) dye. The degradation performance of CP-MgONPs showed over 94% photocatalytic degradation efficiency of acid orange 8 (AO-8) dyes within a short time.

**Conclusions:**

Outcomes of this research signify that biogenic CP-MgONPs may be advantageous at low concentrations, with positive environmental impacts.

## 1. Introduction

Nowadays, research has its emphasis on green chemistry and necessitates more work to incorporate sustainable practices in order to manufacture desired products and eventually minimize and eliminate waste materials generated [1]. Green synthesis has garnered enormous scrutiny as an efficient, enduring, and environmental benign approach for creating plethora of nanomaterials. The industrial revolution of the 21^st^ century is said to have been sparked by the marvels of contemporary medicine [2]. The use of nanomaterial loaded with pharmaceuticals such as phytochemicals, vitamins, and hyaluronic acids has emerged as an intriguing substitute [3]. Plant and plant waste are valuable source of myriad bioactive compounds including flavonoids, terpenoids, alkaloids, steroids, carbohydrates, and acids [4]. These plant phytoconstituents act as capping agents and natural bio-reductants of metal/metal oxide ions, thereby sterically stabilizing nanomaterials by reduction through direct molecular interaction [5]. Scientific analysis has shown that large volumes of bio-wastes produced from fruit and vegetable peels, which are a rich source of several biocomponents, are neglected, ultimately putting market vendors in risky circumstances [6]. A feasible approach of reducing bio-waste can be established through its application in the production of advantageous nanomaterials [7]. The uprising of metal-based nanomaterials represents a major advancement in nanotechnology towards the development of superior commodities. Recently, iron, silver, and gold nanoparticles with potent antibacterial and water remediation properties have been successfully prepared from peels of fruits and vegetables as well as their bagasse [8]. However, carbon nanostructures developed from these agricultural wastes have been used in the industrial sector to create materials with excellent electrical conductivity and photocatalytic activity, which are used in the production of green fuels, energy storage devices, and water purification systems [9]. The most valuable metal-based nanomaterials prepared by the green approaches include silver, gold, zinc, magnesium, copper, iron, and their respective oxides [10]. The significant interest in them emanates from their admissible physicochemical features and wide variety of agricultural, food, medical, and industrial use [11]. Ag, Au, Cu, and ZnO nanoparticles were studied extensively in pharmaceutical industry for their versatile physicochemical features and diverse biomedical applications [12]. However, their accumulation in the body makes them often associated with substantial toxicity risk. Unlike all other nanoparticles, MgO nanoparticles are extremely biocompatible, commercially feasible, and stable in harsh environments [13]. MgONPs have distinctive features including a high refractive index, strong corrosion resistance, and superior thermal and poor electrical conductivity [14]. It also has a persistent interaction with botanical constituents to generate nanoparticles due to high surface area and highly reactive edges. These characteristics give MgONPs access to various domains, including catalysis, electronics, additives, ceramics, photochemical products, drug synthesis, agricultural commodities, sensors, antimicrobial materials, and adsorbents [15]. They also function exceptionally well as adsorbents for eliminating chemical contaminants from wastewater and this benefit increases with decreasing MgO size [16]. Their unique biocompatibility and physicochemical stability have made them an excellent nanocarrier. They benefit from having a high degree of ionization, photocatalytic abilities, and efficient endurance to elevated temperatures [17]. Notably, the FDA has recognized MgONPs as a safe, convenient alternative with strong antibacterial properties [18].

Conventionally, synthesis of MgONPs has been accomplished by a variety of physical and chemical techniques, including chemical precipitation, thermal decomposition, sol-gel, chemical vapor deposition, and combustion [19]. These techniques often need several processing stages, pH control, high temperature and pressure, pricey equipment, and hazardous chemicals and generate numerous side products that may be harmful to the ecosystem. Thus, there is a constant need to develop greener way that is energy-efficient, economical, and ecologically friendly to avoid hazardous chemicals in nanoparticle synthesis [20]. To triumph over these challenges, natural products (botanicals, bacteria, fungi, lichens, agricultural waste, etc.) offer great resources for the phytogenic production of metal/metal oxide nanomaterials. The accessibility, environmental friendliness, safety, and overall nontoxicity of biomasses of natural products make them a desirable choice for the fabrication of MgO nanoparticles [21]. Numerous types of phytochemicals are contained in these natural products that function as a reductants and chemical stabilizers in the formation of metal/metal oxide nanostructures [15].

*Citrus medica* (citron) has commercially been cultivated in many countries especially in tropical and subtropical regions. Its fruit peel, pulp, and seeds have applications in traditional medicine to cure abdominal colic, digestive issues, and piles. Citron fruits are akin to large lemons in appearance, with a thick skin and little pulp content. The culinary industry uses it extensively in a variety of items, such as cakes, confections, marmalades, soft drinks, and dairy products [22]. However, the processing of citron fruit for these items produces larger percentage of byproducts (peels, seeds, leaves, and stems) compared to that achieved of temperate fruits, which are often discarded as agricultural trash [23]. About one third of the fruit that is removed after harvesting is made up of citron peels.. The literature survey has revealed that citron peel contains a considerable amount of bioactive components, including phenolic, flavonoids essential oil (EO), carotenoids, and compounds with diverse cross section of functional groups (carbonyl, carboxyl, ketones, and aldehyde groups) that can be exploited for different purposes [24]. Also, the peels of citron have exhibited highest antioxidant and antibacterial activity in contrast to seeds and pulp [25]. The agricultural industry can profit from these waste streams since these bioactive compounds can be effective reductants, stabilizers, and capping agents during the synthesis of metal oxide nanomaterials. Previous research work has addressed the formation of copper oxide, gold, silver, and selenium nanomaterials from the juice, seed oil, fruit waste peel, and leaf of *C. medica*, respectively [26–30]. Hence, considering the rich chemoprofile and medicinal properties of citron peel, it was postulated that using peels to make nanoparticles would produce nanomaterials with medicinal attributes that would be beneficial for the biological system. The present study was intended to biosynthesize MgONPs using aqueous extract of citron peel, characterize them, and evaluate their antibacterial and anticancer activities. In addition, the presynthesized CP-MgONPs were also examined for photocatalytic ability.

## 2. Materials and Methods

### 2.1. Chemicals and Reagents

Magnesium nitrate hexahydrate (Mg(NO_3_)_2_·6H_2_O, ≥99%), sodium hydroxide (NaOH, ≥98%), ethanol (EtOH, 95%), dimethyl sulfoxide (DMSO, ≥99%), Soy broth (TSB), Mueller–Hinton Agar plates, Augmentin, Gentamicin, Norfloxacin (C_16_H_18_FN_3_O_3_, ≥98%), Amikacin (C_22_H_43_N_5_O_13_), Tetracycline (C_22_H_24_N_2_O_8_·xH_2_O, 98–102.0%), Cefotaxime (C_16_H_16_N_5_NaO_7_S_2_), Ciprofloxacin (C_17_H_18_FN_3_O_3_, ≥98%), Ampicillin (C_16_H_19_N_3_O_4_S, 96–102.0%), MTT (3-(4,5-Dimethylthiazol-2-yl)-2,5-diphenyltetrazolium bromide), Dulbecco's modified Eagle medium (DMEM), Lipid peroxidation (LPO), Glutathione (GSH), Glutathione peroxidase (GPx), Superoxide dismutases (SOD), Catalase, Ethidium bromide (EB) dye, Rhodamine 123 (≥85%), Paraformaldehyde (PFA, 95%), Propidium iodide (PI, ≥94%), 4′, 6-Diamidino-2-phenylindole dihydrochloride (DAPI, ≥98%), Protease cocktail inhibitor, Nonfat milk powder (5%, pH, 7.3), Caspase 3, 8, 9 (≥95%), *β*-Actin, Prolamin, cleaved Poly ADP-ribose polymerase (PARP), cleaved Lamin, Horseradish peroxidase (HRP, ≥250 units/mg), 3,3′,5,5′-Tetramethylbenzidine (TMB, ≥98%), hydrogen peroxide (H_2_O_2_), and Acid orange-8 (AO-8 ∼60%) used in this research were acquired from Sigma-Aldrich (Hamburg, Germany).

### 2.2. Microorganisms and Cell Culture

Six pathogens including *B. cereus* (*ATCC 10876*), *E. coli* (*ATCC 25922*), *K. pneumoniae* (*ATCC 13883*), *P. aeruginosa* (*ATCC 27853*), *S. aureus* (*ATCC 25923*), and *S. pneumoniae* (*ATCC 27336*) were used to examine the antibacterial activity of presynthesized CP-MgONPs. The anticancer potential of formed CP-MgONPs was tested on human epidermoid carcinoma (Hep2), human colon adenocarcinoma (COLO 205), and neuroblastoma (SH-SY5Y) cancer cells. Both microorganisms and cancer cell lines were supplied by King Khalid Hospital (KKHU), Riyadh, Saudi Arabia (SA). The cancer cells were raised at 37°C in a moist environment (95% air, 5% CO_2_) in DMEM comprised of glucose (1000 mg/L) and L-glutamine (1000 mg/L) supplemented with penicillin G (100 units/mL), fetal bovine serum (FBS, 10%), and streptomycin sulfate (0.1 mg·mL^−1^).

### 2.3. Botanical Material and Extraction

Peels from fresh citrons (*Citrus medica*) were gathered from local juice vender after the juice extraction, Riyadh, Saudi Arabia, in March 2021. The peels were sliced into tiny pieces (∼0.5 cm) after being properly rinsed multiple times with tap water to get rid of the dust and debris. 100 g cut pieces of fresh citron peels were soaked in 1.5 L deionized water in a 2 L conical flask and heated up to ebullition under vigorous stirring. Heating was stopped once the solution reached the boiling point and was strained through the filter paper (Whatman No. 1) after cooling to remove the peel pieces. Afterward, a second filtration step of mixture was conducted under vacuum using a MF-MilliPoreTM membrane filter with 0.22 *μ*m pore size. The citron peel extraction has been repeated twice under comparable conditions. After pooling, the volume of aqueous extract was minimized on a rotavapour at 45°C under reduced pressure. The resultant pale yellow solution was collected in a glass media bottle and stored at −20°C till another experimental usage.

### 2.4. Green Biosynthesis of MgO Nanoparticles

The biogenic MgO nanoparticles were prepared by obeying Sharma et al. protocol with a few minor modifications [31]. To put it briefly, 100 mL of aqueous citron peel extract was infused with 150 mL of freshly prepared magnesium nitrate (1.0 mM) solution, followed by dropwise addition of 10 mL of NaOH (1.0 M) at 80°C, and the mixture was continuously stirred for 6 h on a magnetic stirrer at 600 rpm. The pale yellow to yellowish-brown color transition functioned as a signal for the production of nanoparticles. The precipitate of generated CP-MgONPs was then collected by the centrifugation of reaction mixture at 5000 rpm for 15 min. The dried CP-MgONPs were obtained by calcining the precipitate at 400°C in a furnace after it had been repeatedly cleaned of impurities with ethanol.

### 2.5. Characterization of MgO Nanoparticles

Various modern strategies were employed for the characterization and identification of presynthesized CP-MgONPs. The formation of CP-MgONPs was initially carried out by visual observation based on color change. The optical characteristic of synthesized CP-MgONPs was studied using UV-Vis absorption spectrophotometer (SM 7504 Uniscope, China) between 200 and 800 nm wavelength. The existence of functional moieties in the biosynthesized CP-MgONPs was performed by Fourier transform infrared (FTIR) spectroscopy with a 4000 to 400 cm^−1^ scanning range (FTIR: Nicolet™ iS50, Thermo Fisher Scientific, USA). Crystallinity and phase purity of the produced nanoparticles were ascertained from their X-ray diffraction (XRD) pattern over 2*θ* range of 20−80° (XRD, Rigaku DMAX-IIIC, Texas, USA) fitted with Cu K*α* source and *λ* = 1.54056 Å at room temperature. Zeta potential measurements were measured using a particle size analyzer that relies on laser scattering light (NS 3000, Zetasizer, Malvern Instruments Ltd, Malvern, UK). The microstructure, surface topography, size, and elemental content of the CP-MgONPs were examined using a scanning electron microscope (SEM: JSM-7660F JEOL, Yokogushi, Japan) outfitted with an energy-dispersive X-ray (EDX) analyzer at a 15 kV accelerating voltage. The transmission electron microscopy with a point resolution of 0.45 nm (TEM JEM-ARM200F-G) at a 200 kV accelerating voltage was applied to measure the particle size and their dissemination using ImageJ software. The thermal integrity of CP-MgONPs was accessed by thermogravimetric analysis using TA instrument Q series™ thermal analyzer (Q600-DSC/TGA, Champaign County, USA).

### 2.6. Antimicrobial Effect of the Biogenic CP-MgONPs

Six pathogenic microorganisms that are often related to food diseases and nosocomial infections in humans were applied to test the antimicrobial potential of the presynthesized CP-MgONPs. Tryptic soy broth (TSB) was the medium used to subculture each selected pure bacterial isolates, and the cultures were thereafter aerobically incubated for 24 h at 37°C. A 10^7^ CFU/mL of viable cell with 0.4–0.5 optical density of bacterial cultures was achieved by adjusting absorbance at 640 nm using a spectrophotometer.

#### 2.6.1. Antibiogram Evaluation of Bacterial Strains

In this investigation, the microbial strains were screened using the Kirby–Bauer technique on Mueller–Hinton agar plates as an internal control against standard antibiotics. Gentamicin (30 *μ*g), Augmentin (30 *μ*g), Amikacin (30 *μ*g), Ampicillin (10 *μ*g), Tetracycline (30 *μ*g), Cefotaxime (30 *μ*g), Ciprofloxacin (5 *μ*g), and Norfloxacin (10 *μ*g) were employed as antibiotic discs. The agar plates were spread-plated with overnight preadjusted bacterial cultures (10^6^ cfu/mL), and sterile forceps were used to evenly space various antibiotic discs on the inoculated agar. A 24 h aerobic incubation period was then applied to the each plate at 37°C. The readings for the antibiotic inhibition zone diameter (AIZD) were determined and expressed in millimeters (mm). Standard reference values have been established for classifying bacterial isolates as susceptible, moderately resistant, or resistant to a certain antibiotic based on the AIZD data [32].

#### 2.6.2. Antibacterial Potential of Biogenic CP-MgONPs

The antibacterial potency of presynthesized CP-MgONPs has been tested with the agar well diffusion assay by adopting previously reported Ifeanyichukwu et al. procedure with minor alteration [33]. In brief, 100 *μ*L of aliquots of bacterial culture (10^6^ cfu/mL) was laid on agar plates to form a smooth lawn and allowed to stand for 10 min. A hole (well) with a diameter of 8 mm was punched using a sterilized cork borer in the media containing agar plate and labeled. After then, 50 *μ*L aliquot of nanoparticle solution (25 *μ*g·mL^−1^) was added into each well on all plate and allowed to stand in the biosafety chamber for 1 h to ensure that the test samples are evenly spaced within the agar, followed by incubation for 24 h at 37°C. Millimeters (mm) were applied to express the diameter of the growth inhibition zone of bacteria and noted. The test was executed in triplicates and the Statistical Package for the Social Sciences (SPSS, 20.0 software version) was adapted to statistically estimate the pooled data. Gentamicin was administrated as positive control and dimethyl sulfoxide (DMSO) was negative control for the study.

#### 2.6.3. Minimum Inhibitory Concentration (MIC) Determination of Biogenic CP-MgONPs

The lowest possible amount of an antimicrobial agent required for preventing microbial growth after 24 h of incubation period is known as the MIC [34]. The effectiveness of biogenic CP-MgONPs in restricting pathogenic growth was assessed by selecting the optimal concentration of CP-MgONPs that had a significant antibacterial effect at 2500 *μ*g·mL^−1^. This concentration was then used for determining the MIC in 96-well plates by employing the microbroth dilution procedure [35]. The initial concentration (2500 *μ*g·mL^−1^) of nanoparticles was aseptically diluted two-fold by pouring 100 *μ*L of the sterile CP-MgONPs into 100 *μ*L of tryptic soy broth in a well plate to establish a concentration of 1250 *μ*g·mL^−1^. The entire procedure was repeated multiple times to acquire different doses (625 *μ*g·mL^−1^, 312 *μ*g·mL^−1^), and a 312-fold dilution of initial CP-MgONPs concentration (2500 *μ*g·mL^−1^) was created aseptically by pouring 100 *μ*L of the standardized suspension of bacteria (10^6^ cfu/mL, OD_640nm_ = 0.1) into the both plates and wells, which were then incubated for whole day at 37°C. After overnight incubation, the OD of the plates has been determined at 640 nm absorbance wavelength on a microplate reader, to establish the lowest inhibitory concentration required to prevent the bacterial cell growth.

#### 2.6.4. Time-Kill Kinetics Assay

Time-kill kinetics assays serve a purpose in understanding the interaction that occurs between microorganisms and antimicrobials. This assay demonstrates a dose or time-dependent test impact of antimicrobials on various pathogens. It differentiates between bacteriostatic and bactericidal antimicrobial drugs. The time-kill kinetics measurements of CP-MgONPs towards the bacteria under investigation were assessed using an altered time-kill kinetics procedure [36]. The fabricated CP-MgONPs were reconstituted in DMSO to achieve various concentrations (2.5 − 0.01 mg·mL^−1^) in order to estimate the time. The well plates containing 100 *μ*L of TSB were loaded with various doses of CP-MgONPs, followed by addition of 100 *μ*L of bacterial culture into the each wells and incubated at 37°C. After every hour, OD was measured and noted. A graph plotted between optical density *vs* time was used to assess the killing time kinetics that represent the cell response to the nanoparticle exposure.

#### 2.6.5. Evaluation of Morphological Changes in *E. coli* and *S. aureus*

The morphological alterations of the *E. coli* and *S. aureus* treated with presynthesized CP-MgONPs were observed by SEM. The CP-MgONPs-treated bacterial strains were segmented into pieces (5–10 mm) and they were then mounted for 1 h on a glass slide in phosphate-buffered saline solution with 3% glutaraldehyde. The treated tissues were then dried using carbon dioxide and ethanol. The dehydrated tissues were positioned on the aluminium stubs using a gold-palladium coated silver pan and morphology of the bacteria was observed at a 15 kV voltage of acceleration under SEM.

### 2.7. Cytotoxicity Evaluation of the Biogenic CP-MgONPs

The antiproliferative behaviour of biogenic CP-MgONPs on Hep2, OLO 205, and SH-SY5Y cancer cells was examined by MTT assay adapted from previously reported method with minor modification [37]. Briefly, cells were cultured (1 × 10^4^ cells/well) with 80% confluence for 24 h in a 96-well plate. After seeding for 24 h period, cells went through exposure to different concentrations of CP-MgONPs (25, 50, 75, 100, 125, and 150 *μ*g·mL^−1^) and incubated for extra 24 h. Then, each well was filled with 10 *μ*L of MTT (5 mg·mL^−1^ in PBS) solution, and the mixture was incubated for 4 h more at 37°C to develop the color. The reaction was stopped and the blue formazan crystals were dissolved by adding an equivalent amount of DMSO to the every well. Finally, a microplate reader was implemented to record the absorbance of the reaction mixture at 570 nm wavelength. The cell viability was determined by applying the given equation and IC_50_ values were then computed.

(1) $$\begin{array}{c} \text{Viability Percentage}=\left(\frac{\text{Absorption }\left(\text{test}\right)}{\text{Absorption }\left(\text{control}\right)}\right)\times 100. \end{array}$$

#### 2.7.1. Cytomorphological Analysis

The vulnerable, cultivated Hep2 cells were planted at a 1 × 10^5^ cells/well density onto a 12- well chamber plate and kept in a humid environment with continuous flow of CO_2_ (5%) for 12 h at 37°C. The seeded cells were then administrated with CP-MgONPs (100 *μ*g·mL^−1^ in distilled water) and incubated for 24 h. The PBS was utilized twice to rinse cells after incubation and cytomorphological alterations in treated and untreated cells were seen and captured on camera under phase-contrast inverted microscope at 24 and 48 h.

#### 2.7.2. Acridine Orange/Ethidium Bromide (AO/EB) Staining

The degree of apoptosis induced by CP-MgONPs in Hep2 cancerous cells was evaluated by dual AO/EB fluorescent labelling [38]. The cultivated Hep2 cells (1 × 10^5^ cells/well) were seeded in a 12-well plate and placed in a humid environment at 37°C with a steady supply of CO_2_ (5%) for 12 h. Then, the seeded cells received treatment with CP-MgONPs along with their corresponding IC_50_ values and incubated for an entire day at 37°C. Later, the cells were exposed to 50 *μ*L of an AO/EB dye mixture and were monitored under fluorescence microscope to detect any signs of apoptotic cell death.

#### 2.7.3. Intracellular ROS Detection and Quantification

ROS was assessed by obeying the procedure described by Wang and Roper [39]. Briefly, 6 well plates were plated with cultured Hep2 cells and then CP-MgONPs at an IC_50_ concentration were administrated for an entire day. Afterward, 10 *μ*M of DCFH-DA solution was poured into the treated and untreated seeded cells and kept for incubation for half an hour at 37°C. The extent of production of ROS in treated and untreated cells was determined through a fluorescent spectrophotometer. Subsequently, trypsinized CP-MgONPs-treated cancer cells were collected separately in Eppendorf tubes wrapped in aluminium foil for the estimation of ROS. The cells were then administered with 25 M of DCFH-DA solution and allowed to incubate for 45 min at 37°C. The fluorescence intensity in relation to excitation and emission wavelengths was measured by Fluorolog-FL3-11 spectrofluorometer.

#### 2.7.4. Oxidative Stress Factors

After being cultured in flasks (75 cm^2^), the Hep2 cells were exposed for 24 h to CP-MgONPs at an IC_50_ dose. Cells were collected in ice-cold PBS at 4°C after being treated and rinsed with PBS. The cells were then lysed with lysis buffer that included Na_2_EDTA (1 mM), Triton (1%), NaCl (150 mM), Tris-HCl (20 mM, pH 7.5), and Na_4_O_7_P_2_ (2.5 mM). The supernatant from the centrifugation of the lysed cell homogenate for 10 min at 10,000 rpm was applied to measure the oxidative stress markers. The estimation of many indicators of liver oxidative stress including LPO, GPx, SOD, catalase, and nonenzymatic antioxidant GSH was performed by obeying standard procedures [40].

#### 2.7.5. Evaluation of Mitochondrial Membrane Potential (MMP or Δ*Ψm*)

Oxidative stress results in the inactivation of the mitochondrial membrane potency and consequent loss of function. The mitochondrial membrane potential loss in Hep2 cells treated with CP-MgONPs was evaluated using rhodamine 123, a fluorescent indicator that preferentially deposits within the mitochondria based on membrane potential [41]. In brief, Hep2 cells (5 × 10^5^ cells/well) have been introduced onto 6 well plates and incubated for 12 h at 37°C under a constant CO_2_ (5%) flow. Later, the Hep2 cells were exposed for 24 h to CP-MgONPs at an IC_50_ concentration. Following PBS wash, cells were fixed for 10 min in 4% paraformaldehyde and 70% ethanol. Subsequently, 50 *μ*L solution of rhodamine 123 (10 *μ*g/mL) was poured into each well and held for half an hour. After repeated PBS washes to remove excessive dye, cells were examined at 20 × resolution on a fluorescence microscope. Furthermore, the treated and untreated cells were trypsinized for quantification and separated into in Eppendorf tubes wrapped in aluminium foil, followed by addition of rhodamine 123 and keeping the mixture at 37°C for 45 min. Finally, the fluorescence intensity and its associated wavelengths of excitation and emission were then recorded on a FL311-Fluorolog spectrofluorometer.

#### 2.7.6. Propidium Iodide (PI) and 4′, 6-Diamidino-2-phenylindole Dihydrochloride (DAPI) Staining

The apoptotic alterations such as fragmentation/condensation in the cell nucleus were detected by applying PI staining. In brief, cultured Hep2 cells (1 × 10^5^ cells/well) were plated onto 6 well plates and left overnight to incubate. The cells were then exposed to CP-MgONPs at its IC_50_ concentration and incubated further for 24 h at 37°C in a humid environment. After CP-MgONPs treatment, the Hep2 cells were rinsed with PBS and anchored in 4% paraformaldehyde and 70% ethanol, respectively. Thereafter, cells were nurtured with DAPI solution at 37°C for 10 min. The cells were stained with PI solution for 10 min after being cleaned with PBS. Lastly, the stained cells were then repeatedly rinsed with PBS to eliminate the excessive PI, and they were examined at a 20 × resolution under a microscope [42].

#### 2.7.7. Western Blot Analysis

The protein identification was conducted by Western blotting. The Hep2 cells were treated with varied concentration (20, 40, and 60 *μ*g·mL^−1^) of biogenic CP-MgONPs and were then held for 24 h in a humid environment with steady supply of CO2 (5%) at 37°C. The cells were then separated, twice rinsed with ice-cold PBS, and lysed using protease cocktail inhibitor with lysis buffer after being incubated for an entire day. The protein content of the supernatant was analyzed by the Lowry procedure after centrifugation of lysates for 10 min at 8000 rpm at 4°C. Equal sample concentrations were separated by applying 12% sodium dodecyl sulfate-polyacrylamide gel (SDS-PAGE) through electrophoresis. Proteins that had been separated were laid on nitrocellulose membranes, which were subsequently blocked with 5% skim milk solution for duration of one hour. After multiple PBS washing, the main antibodies (active caspase 3, 8, 9, *β*-actin, prolamin, cleaved PARP, and cleaved lamin) were poured at 1 : 1000 (v/v) ratio and incubated for a whole night at 4°C. Later, the secondary antibody coupled to horseradish peroxidase (HRP) was applied and left aside at room temperature for 2 h after the primary antibodies were removed. The enhanced chemiluminescence (ECL) solution was used to visualize the protein band, and ImageJ software was used for quantification [43].

### 2.8. Photocatalytic Activity

Heterogeneous photocatalysis was used to assess the potential of CP-MgONPs for the removal of dye from wastewaters. Acid orange-8 (AO-8) dye was used to examine the photocatalytic efficacy of the CP-MgONPs. A homemade photoreactor was used to conduct the photocatalytic process, and it was furnished with a 250 W Osram mercury lamp with high pressure to provide UV radiation. A glass Pyrex beaker with a magnetic stirring rod constituted the reactor and the light source is situated over the beaker. The Pyrex glass beaker and the light were placed 20 cm apart. The photocatalytic reactor was enclosed in a box to avoid lethal radiation. Briefly, 80 mg of CP-MgONPs was dispersed in 100 mL aqueous solution of AO-8 2.50 mg/250 mL. Prior to UV illumination exposure, the suspension was magnetically agitated in the dark for 30 min to ensure that the mixture was properly homogeneous and that the absorption equilibrium was reached. After certain time intervals of irradiation, a given volume (4 mL) of the reaction mixture has been gathered and filtered through 0.22 *μ*m Millipore membranes and then the solution was centrifuged for 10 min at 5000 rpm to extract photocatalyst particle suspensions. The irradiated AO-8 solution was eventually examined using UV-Vis spectrophotometer, scanned in 200–800 nm UV-visible range and then monitored at *λ*_max_ 480–490 nm wavelength [44]. The following calculation was applied to ascertain the percentage of photocatalytic degradation:

(2) $$\begin{array}{c} \text{Photodegardation efficiency }\left(\%\right)=\frac{A_{0}-A}{A_{0}}\times 100. \end{array}$$

The degradation's rate constant, *k*, was established utilizing the equation from the first-order plot: ln (*A*_0_/*A*) = *kt*, where initial dye absorbance is represented by *A*_0_, and dye solution absorbance is denoted by *A* after irradiation of UV light.

### 2.9. Statistical Analysis

The data disclosed were based on the means of three independent replicates. The data were statistically estimated by statistical software program SPSS v17. The analysis of the average variation among the treatments was done with a *t*-test or analysis of variance (ANOVA). The HSD Tukey test then ensued at *p* < 0.05.

## 3. Results and Discussion

### 3.1. Spectrophotometric Characterization of CP-MgONPs

**Figure 1:**
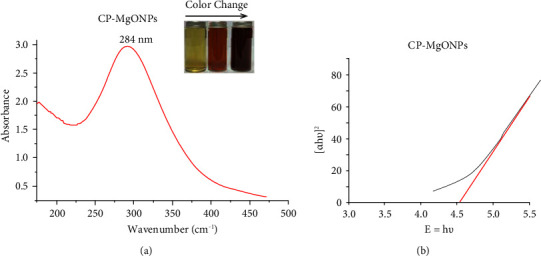
(a) UV-Vis absorption spectrum and (b) band gap energy of CP-MgONPs.

The phytochemical components found in the citron waste peels were recognized as a helpful ingredient for producing CP-MgONPs. Citron peel-mediated magnesium oxide nanoparticles (CP-MgONPs) were created when the aqueous magnesium ions were reduced by the citron peel extract. The color alteration from pale yellow to yellowish-brown was the first evidence of the formation of presynthesized CP-MgONPs during the integration of aqueous citron peel extract and Mg(NO_3_)_2_.6H_2_O. The triggering of vibrations of surface plasmon led to emergence of yellowish-brown, which is a hallmark of MgONPs having *ʎ*_max_ values between 260 and 300 nm in the visible domain [45]. The size, morphologies, nature, well distribution, particle-to-particle distance, and the surrounding medium of the formed nanoparticles all had a significant impact on the SPR absorbance [46]. The biosynthesized MgONPs have tendency to be smaller in size at less than 300 nm SPR values; however, the anisotropy increases with SPR over 300 nm, as described by Jeevanandam et al. [47]. In the current study, the optical characteristics of citron peel-mediated synthesized CP-MgONPs were measured between 200 and 800 nm wavelength range and UV-Vis spectra of CP-MgONPs exhibited a sharp plasmon resonance band at 284 nm (Figure 1(a)), indicating the presence of nanoscale particles. The findings presented were consistent with those published by Hassan et al. [48], Abdallah et al. [49], and Nguyen et al. [50]. These investigations demonstrated that the most significant bands of MgONPs prepared from *Rhizopus oryzae*, *Tecoma stans* (L.), and *Rosmarinus officinalis* L occurred at 282, 281 nm, and 250 nm, respectively. Further, there was a noticeable absorption band at 290 nm in chemically synthesized MgONPs [51], suggesting the bioactive components found in the citron extract were effective at reducing, capping, and stabilizing. The absorption spectra of biogenic NPs were calculated by applying Tauc's relation, (*αhν*)^2^ = *B*(*hυ*-*Eg*), to determine the band gap energy of CP-MgONPs. In this equation “*α*” denoted the absorption coefficient and “*B*” represents a constant. The term *hѵ* represents the photon energy and *Eg* signifies the band energy. The band gap of presynthesized CP-MgONPs was determined as the linear portion of the curve between (*αhν*)^2^ and (*hν*). The estimated amount of band gap energy was 4.62 eV for CP-MgONPs as depicted in Figure 1(b). The value of band gap is aligning with previously reported literature, and it might be associated with the quantum confinement effect [52].

In this investigation, an extracellular approach was employed since it was more feasible and resulting nanomaterials were easy to purify. Numerous components found in the hydroextract of citron peels function as reductants and the nanoparticle biosynthesis was conducted in alkaline medium as it promotes the reduction ability of functional moieties and avoids nanoparticle aggregation [53]. It further aids in the stabilization and capping of nanoparticles by interacting with the amine functions of proteins that are attached to the surfaces and their residual amino acids.

**Figure 2:**
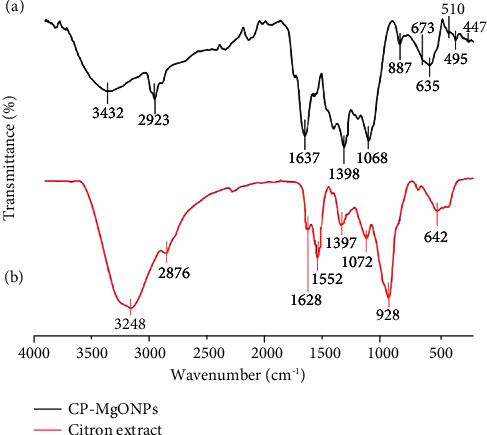
FTIR spectrum of (a) CP-MgONPs and (b) citron peel extract in the wavelength range of 4000 − 400 cm^−1^.

The double function of the plant biomass as a reductant and capping agent of the anchored functional moieties on the CP-MgONPs surface was recognized by FTIR analysis in the wavelength range of 4000 − 400 cm^−1^ (Figures 2(a) and 2(b)). The FTIR spectrum of biogenic CP-MgONPs exhibited bands at 3432, 2923, 1637, 1398, 1346, 1068, 673, and 887 cm^−1^ (Figure 2(a)) that correspond to O-H-stretching for alcohols, C-H stretching for alkane, C=C stretching for di-substituted (cis), O-H bending for carboxylic acid, O-H bending for alcohols, C-O stretching for primary alcohols, strong C=C bending for di-substituted (cis) alkenes, and strong C=C bending for vinylidene (alkene), respectively, suggesting the existence of several bioactive metabolites such as phenolics, flavonoids, tannins, and carbohydrates adsorbed on the surface of NPs [54]. The active functional moieties in these metabolites are essential for the preparation and stability of CP-MgONPs. Moreover, the FTIR spectrum of CP-MgONPs also showed two absorption peaks at 635 cm^−1^ and 495 cm^−1^ that were associated with the MgO vibrations, suggesting the MgO nanoparticle formation [55]. Various peaks were observed at 447, 510, 582, and 670 cm^−1^ which were linked to the CP-MgONPs vibrations. The shift observed in various peaks of biosynthesized CP-MgONPs in contrast to citron peel extract may be due to interaction of MgO vibrations with functional groups of citron peels extracts.

**Figure 3:**
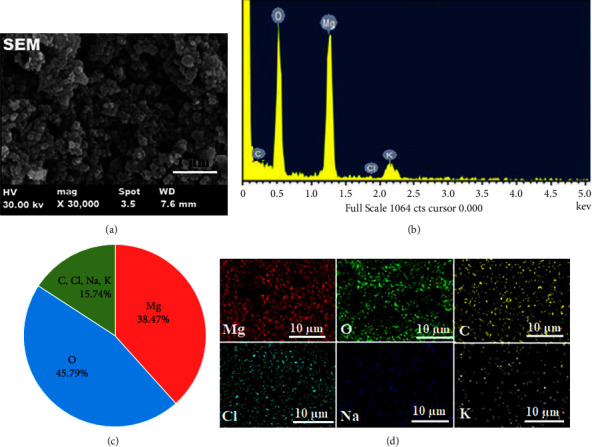
(a) SEM at ×30,000 magnification, (b) EDX, (c) pie chart, and (d) EDS mapping spectrum of as-synthesized CP-MgONPs.

Generally, the activity profile of nanoparticles is often linked to various features such as size, shape, and distribution. Thus, it has significance to assess the size and morphology of the formed nanoparticle. The SEM micrograph of biosynthesized CP-MgONPs displayed polyhedral shape with agglomeration that may be possibly caused by electrostatic attraction of MgONPs as described by Pugazhendhi et al. [56]. The formed nanoparticle has an average diameter of 38.7 nm, and its particle size dispersion is shown in Figure 3(a). It is intriguing to note that almost all the CP-MgONPs were uniformly disseminated and encompassed by polyphenol biocomponent layer, providing evidence that the CP-MgONPs were capped and distributed by the biocomponents present in citron peel extract. The elemental composition of CP-MgONPs was examined using energy scattering spectroscopy (EDX). The EDX chart demonstrated the excellent purity of the presynthesized CP-MgONPs containing Mg and O ions, demonstrating that CP-MgONPs had been successfully produced by exploiting the metabolites of citron peel aqueous extract. Also, the occurrence of two peaks for Mg and O at 0.5–1.5 keV range of bending energies supports the successful MgO formation (Figure 3(b)). The EDX spectrum showed prominent signals for magnesium and oxygen with mean percentage of 38.47% and 45.79%, respectively. The existence of carbon (C) peak signal with mean 15.74% as well as weaker peaks for hydrogen (H), chloride (Cl), sodium (Na), and potassium (K) might be due to the presence of bioactive components binding to the surface of presynthesized CP-MgONPs (Figure 3(c)). In fact, the production of magnesium oxide nanomaterials was significantly affected by high concentration of flavonoids. The elemental composition and their distribution in the presynthesized CP-MgONPs were examined by X-ray energy-dispersive spectroscopy mapping (EDS). The elemental mapping presented in Figure 3(d) confirmed the presence of C, Cl, Na, and K in the CP-MgONPs sample. The distribution of each element and their mapping suggested that they were uniformly distributed due to high compatibility between the components.

**Figure 4:**
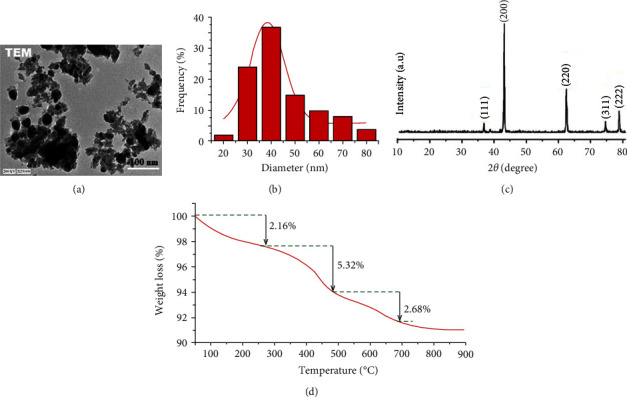
(a) TEM image at ×25000. (b) Size distribution. (c) XRD. (d) Thermogravimetric analysis of biogenic CP-MgONPs.

The surface morphological traits and size formation of the formed CP-MgONPs were analyzed by HR-TEM. The HR-TEM micrograph of CP-MgONPs clearly exhibited the polyhedral shape of presynthesized CP-MgONPs at ×25000 magnifications (Figure 4(a)), in agreement with previous study [18]. The nanometer size of presynthesized CP-MgONPs appeared with size 34.45–52.13 nm range under HR-TEM at 100 nm (Figure 4(b)), which is similar to SEM observation. The area selected for electron diffraction pattern displays rings assigned to CP-MgONPs crystal planes. However, the crystalline structure and purity of phase of the biogenic CP-MgONPs have been established by the XRD technique. The diffraction peaks observed in diffractogram confirm the crystallinity of presynthesized CP-MgONPs (Figure 4(c)). The diffraction pattern showed five distinct peaks at 2*θ* = 36.812°, 42.35°, 62.187°, 73.276°, and 78.365° complying with the reflection plane indices of (111), (200), (220), (311), and (222), respectively. The observed peaks illustrated crystallographic structure and were easily assigned to different MgO cubic phase crystal planes devoid of any secondary peak signal, signifying the purity of the presynthesized CP-MgONPs. The intensity and angular location of the peaks were compared with data and matched those in the reference standard file (JCDPS No. 75-0447), confirming the emergence of cubic phase of MgO [57]. The size of biogenic CP-MgONPs can be determined according to sharp XRD peak width (200) positioned at a 2*θ* value of 42.351° by applying Scherrer's equation. The findings revealed that the average crystal size of biogenic CP-MgONPs was 38.7 nm as per XRD analysis. The green accomplished MgONPs prepared with different bioactive metabolites have demonstrated a comparable XRD pattern [48, 58].

Furthermore, the thermal stability and oxygen functionality of the caped green biogenic CP-MgONPs were examined by thermogravimetric analysis (TGA). The thermogram of the CP-MgONPs revealed three identical and constant phases of weight loss (Figure 4(d)). The initial stage of disintegration takes place between ambient temperature and 180°C with a weight loss of 2.16%, indicating that water-containing contaminants have been eliminated from the CP-MgONPs. The removal of water and water-related impurities was symbolized by wide endothermic peak at around 160°C with 2.16% of weight loss, while the 5.32% weight decrease in the second stage was observed between 180 and 552°C and was associated with breakdown of organic molecules (phenolics and flavonoids) that function as capping/reducing agents for the CP-MgONPs. The appearance of two broad peaks at 240 and 365°C suggested that the nature of reaction was endothermic at that point. This outcome was consistent with that of the FTIR spectra, whereas the third and last phase of decomposition involves disintegration of CP-MgONPs at an elevated temperature between 553 and 800°C and results in 2.86% of weight reduction. The percentage purity of biosynthesized CP-MgONPs was estimated to be 92.31% and a comparable TGA range was reported in a previous study for a one-pot crystalline MgONPs synthesis [59].

### 3.2. Antibacterial Activity of Biosynthesized CP-MgONPs

Antibacterial resistance continues to be a major barrier to controlling of infectious diseases in medical systems, animal husbandry, and the food manufacturing sector. The disc diffusion method was employed to evaluate the antibiogram test against six strains of bacteria employed in this investigation, by abiding the recommendations of the Clinical Laboratory Standard Institute (CSLI). The findings were presented by measurements of inhibitory zone diameter in millimeters (mm). A summary of the study on antibiogram testing is provided in Table 1, and Figure S1 (Supplementary data as zone of inhibition for all the test bacteria S1) depicts the visual portrayal of the performed antibiogram test. The antibiogram test outcomes showed that *E. coli*, *B. cereus*, *K. pneumoniae*, *S. aureus*, and *S. pneumonia* were susceptible to Tetracycline, while *P. aeruginosa* exhibited resistance to Tetracycline (Table 1). However, *E. coli*, *K. pneumoniae*, and *S. aureus* were found to be Ampicillin-resistant, whereas *S. pneumoniae* and *B. cereus* were found to be Ampicillin-susceptible. All six strains of pathogen tested in this investigation exhibited sensitivity to the antibiotics Amikacin, Gentamicin, Augmentin, Ciprofloxacin, and Norfloxacin. However, all the applied bacterial strains were resistant to Cefotaxime antibiotic, with the exception of *P. aeruginosa*. Thus, the result revealed that the highly efficient antibiotics were Augmentin, Gentamicin, Amikacin, Ciprofloxacin, and Norfloxacin, whereas Ampicillin and Cefalexin were the most resisted antibiotics (Table 1).

**Figure 5:**
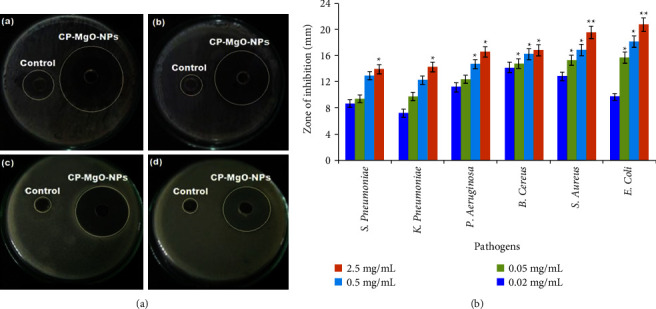
(A) Zone inhibition for (a) *E.coli*, (b) *S. aureus*, (c) *B. cereus*, and (d) *P. aeruginosa* after treatment with biogenic CP-MgONPs at 2500 *μ*g·mL^−1^. (B) Antibacterial effect of biogenic CP-MgO-NPs against tested bacterial strains at various concentrations (2.5, 0.5, 0.05, and 0.02 mg·mL^−1^).

The antibacterial potency of the biosynthesized CP-MgONPs was examined against six microbial strains utilizing the diffusion technique in an agar well. Varied doses (25, 250, 500, and 2500 *μ*g·mL^−1^) of CP-MgONPs were prepared and applied. The antimicrobial effect exerted by biosynthesized CP-MgONPs that inhibit the proliferation of microorganism was discernible as a distinct inhibition zone as depicted in Figure 5(A). Using 2500 *μ*g·mL^−1^ concentration as a standard, the biogenic CP-MgONPs showed significant activity towards all the used pathogenic strains as demonstrated by Table 2. The CP-MgO-NPs showed potent susceptibility to *E. coli* and *S. aureus* with highest inhibition zones of 20.72 ± 0.33 mm and 19.52 ± 0.05 mm, respectively, at 2500 *μ*g·mL^−1^ concentration (Figure 5(A)-(a)-(b)). The formed CP-MgONPs exhibited almost similar antibacterial effects towards *B. cereus* (IZ = 16.78 ± 0.07 mm) and *P. aeruginosa* (IZ = 16.56 ± 0.06 mm) at 2500 *μ*g·mL^−1^ (Figure 5(A)-(c)-(d)). On the other hand, *K. pneumoniae* and *S. pneumonia*e treated with biogenic CP-MgONP showed moderate growth inhibition, with inhibition zones of 14.24 ± 0.09 mm and 13.92 ± 0.01 mm, respectively. Thus, the biosynthesized CP-MgONPs exhibited good sensitivity response towards the both Gram-positive and Gram-negative bacteria at four different doses. The inhibitory diameter zone widened with the rise in CP-MgONPs doses towards each tested strain of bacteria, suggesting the antibacterial effect is dependent on dose.

The observed antibacterial effect might be the result of nanoparticles entering bacterial cells and inhibiting their growth. The small size and morphology of biosynthesized CP-MgONPs as well as the profile of the biologically active components found in the biomass of citron peel extract could be the responsible factors for their penetration and consequently the potent antibacterial effect. Previous studies have also demonstrated that MgONPs have dose-dependent inhibitory effects on wide range of pathogenic bacteria [60–62]. These findings can also be partially attributed to the smallest MgONPs, which have an average diameter of 32–39 nm and have been demonstrated to have potent inhibitory effects against several Gram-negative bacteria in other studies [18]. Considering that the MgONPs that were created before have substantial bactericidal properties as a result of the utilization of *P. guajava* leaves [63], *C. sinensis* leaves [64], *S. costus* roots [65], and so on, it is reasonable to conclude that produced MgONPs are a useful bioresource with antibacterial properties that may find use in the biomedical field and other relevant fields. The precise mechanism behind MgONPs' ability to limit bacterial growth continues to be unresolved, despite the fact that several potential theories have been put forth. Generally, it has been believed that magnesium cations from MgONPs adhered to the negatively charged cell wall of bacteria, which then broke it down, leading to leading denaturing of proteins and ultimately dying of cells [66]. The adherence of magnesium cations to the cell wall conduces to a rise in envelope protein precursors, which in turn promotes a disruption of the proton motive force and inevitably cell demise. It has also been found that MgONPs have the ability to breach the plasma and outer membranes, which depletes intracellular ATP [67]. Another hypothesis that has been proposed is that oxygen and silver interact with sulfhydryl moieties on the cell wall to form R-S-S-R bonds, which stop the cell from breathing and force it to die [68]. Moreover, recent study evidence has indicated that MgONPs may have antimicrobial effects on microorganisms by accelerating the release of ROS, which causes intracellular material to escape through membranes, harming DNA and proteins, and releasing cellular content, and eventually causing cell death[69]. From the results presented in Table 2 and Figure 5, it is apparent that superior antibacterial potential of CP-MgONPs increases with concentration. Similar results were recently reported by Yoon et al. who established that increasing the dosage of formed MgONPs improved their antibacterial activity towards *E. coli* [70]. Das et al. also demonstrated the dose-dependent effectiveness of MgONPs towards the different strains of pathogens [71].

The antibacterial potential of the presynthesized CP-MgONPs was also evaluated by the minimum inhibitory concentration (MIC) method to ascertain the least dosage of CP-MgONPs that can limit the development of bacteria. The outcome demonstrated that 625 *μ*g·mL^−1^ was the lowest concentration of CP-MgONPs required to effectively stop *E. coli* and *S. aureus* from growing, whereas the lowest inhibitory concentration for *B. cereus* and *P. aeruginosa* was achieved at 0.64 *μ*g·mL^−1^. The MIC of CP-MgONPs needed to hinder the growth of *K. pneumoniae* and *S. pneumoniae* was 0.78 *μ*g·mL^−1^ and 0.88 *μ*g·mL^−1^, respectively. However, minimum bactericidal concentration (MBC) for *B. cereus, E. coli*, *K. pneumonia*, *S. aureus*, *S. pneumonia*, and *P. aeruginosa* was found to be 1.74, 1025, 1.96, 1012, 2.11, and 1.89 *μ*g·mL^−1^, respectively. The lowest (most effective) concentration of CP-MgONPs was found 1.74 *μ*g·mL^−1^ in the MBC test against the *B. cereus*. The values of MIC and MBC for biogenic CP-MgONPs are shown in Table 3.

**Figure 6:**
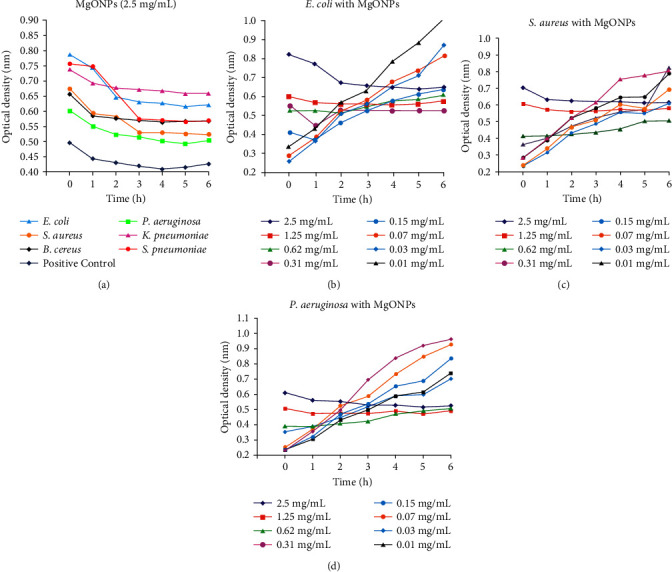
Impact of time on survival of (a) each tested bacterial strain at 2.5 mg·mL^−1^ CP-MgONPs concentration. Impact of time on survival of bacterial strains in 1.25 mg·mL^−1^ CP-MgONPs concentration on (b) *E. coli*, (c) *S. aureus*, and (d) *P. aeruginosa*.

However, the ability of test microbial strains to endure at varied concentrations of biosynthetic CP-MgONPs was assessed by the growth analysis measurements and tracked over the time at 640 nm optical density.  Figure 6(a) illustrates that at 2500 *μ*g·mL^−1^ of CP-MgONPs, the growth of *E. coli, S. aureus, B. cereus,* and *P. aeruginosa* was completely inhibited, but *P. aeruginosa* and *B. cereus* bacterial strains exhibited evidence of growth up until the second hour. The pathogenic strain was still adjusting to the medium when a sharp drop was detected in growth trajectory. An effective inhibition was noticed at 1.25 mg·mL^−1^ concentration. The cellular activity has been noticed in *E. coli* from 0 h to the 1^st^ h. Following that, a spiraling phase was seen between 2^nd^ hour and 4^th^ hour, showing that that CP-MgONPs had disrupted metabolic performance, whereas a stagnant phase was noticed at the 5^th^ hour that ultimately brought about the dying phase at 6^th^ hour (Figure 6(b)) [72].  Figure 6(c), which represents the Gram-positive pathogenic strain *S. aureus*, and Figure 6(d), which represents the Gram-negative pathogenic strain *P. aeruginosa*, respectively, indicate the influence of time on their survival at various concentrations of CP-MgONPs.

**Figure 7:**
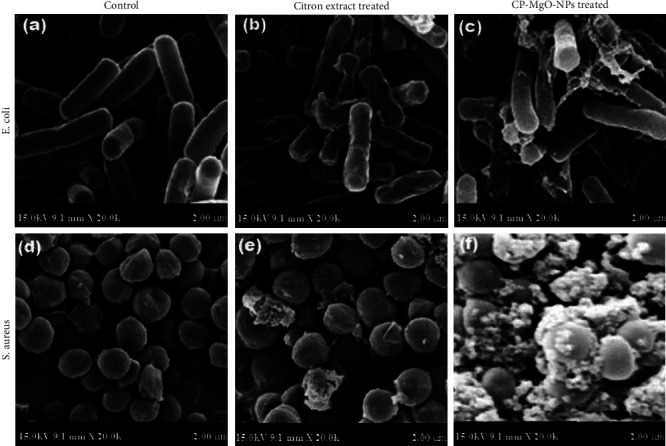
SEM images of (a, d) untreated, (b, e) treated with citron peel extract, and (c, f) treated with biogenic CP-MgO-NPs representing reshaped *E. coli* and *S. aureus*.

The bactericidal effects of CP-MgO-NPs, including cell and membrane damage, may be caused by an electrochemical interaction between LPS and Mg^2+^ ions or oxidative stress brought on by the spontaneous production of ROS and RNS free radicals. Furthermore, the most susceptible strains were discovered to be *E. coli* and *S. aureus* and they were tracked for morphological alterations under SEM (Figure 7). The results obtained revealed that the shape and size of selected bacterial strains were dramatically changed after the treatment with biogenic CP-MgONPs (Figures 7(c) and 7(f)). The presynthesized CP-MgONPs can instantly infiltrate the peptidoglycan layer of the *E. coli* and *S. aureus* cell membrane, rupturing it and allowing components to flow out, killing the pathogenic cell (Figures 7(c) and 7(f)). A positive control for the contrast was chosen to be the pathogenic cells that were not treated (Figures 7(a) and 7(d)).

### 3.3. Anticancer Activity

**Figure 8:**
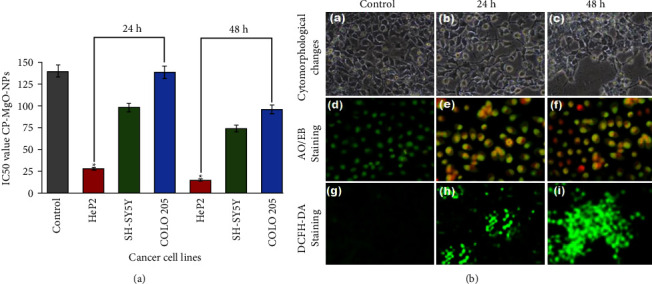
(A) IC_50_ values of CP-MgONPs for Hep2, SH-SY5Y, and COLO 205 cancer cells at multiple points in time of 24 and 48 h. (B) Images of Hep2 cells at ×200 magnification acquired by phase-contrast microscopy: (b, c) alterations in cytomorphology and growth inhibition, (e, f) AO/EB stained cells showing apoptotic, and (h, i) DCFH-DA stained cells showing the ROS level effect of synthesized CP-MgO-NPs on the Hep2 cells, at multiple points in time of 24 and 48 h. (a, d, g) Control.

The anticancer activity of biogenic CP-MgONPs was assessed by MTT assay on three cancer cell lines (Hep2, SH-SY5Y, and COLO 205) and results showed that all the cancer cell lines treated with CP-MgONPs had lower cell viability except Hep2. The IC_50_ values of CP-MgONPs were found to be 28.4 *μ*g·mL^−1^, 98.3 *μ*g·mL^−1^, and 138.4 *μ*g·mL^−1^ for 24 h, while 15.3 *μ*g·mL^−1^, 74 *μ*g·mL^−1^, and 96.1 *μ*g·mL^−1^ for Hep2, SH-SY5Y, and COLO 205 cancer cells, respectively, after 48 h of treatment (Figure 8(A)). Based on the cell viability data, Hep2 cells were found to be most sensitive with low values of IC_50_ and chosen for more research to understand the precise mechanism of action of CP-MgONPs. The Hep2 cells treated with CP-MgONPs showed growth inhibition as well as telltale signs of cell death as cell contraction, chromatin condensation, membrane blebbing, and rounding up of nuclei in the cytomorphological analysis (Figure 8(B)-(a)–(c)). Unlike the untreated group, that exhibited a population of cells with a high cell density and characteristic epithelial shaped cells. The cellular absorption of CP-MgONPs through macro-pinocytosis may have enhanced the formation of ROS, which triggered the apoptotic cascade and provoked cell death as the likely cause of the morphological alterations. On the other hand, the AO/EB double staining clearly demonstrates the late apoptosis by the orange-colored apoptotic bodies that the CP-MgONPs-treated Hep2 cells produced (Figure 8(B)-(d)–(f)).

**Figure 9:**
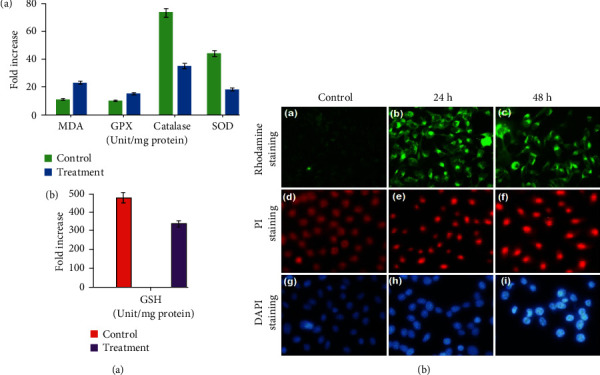
(A) CP-MgONPs effect on the expression levels of (a) MDA, GPX, and Catalase and (b) GSH in CP-MgO-NPs treated Hep2 cancer cell. The mean ± SD of three comparable tests conducted in triplicates was employed to express the data. (B) Fluorescent microscopic photos of Hep2 cell at ×200 magnification: (a, b) rhodamine 123 staining shows disruption mitochondrial membrane potential; PI (e, f) and DAPI (h, i) staining displaying apoptotic bodies and necrotic cell death as consequence of cytotoxicity of biogenic CP-MgO-NPs (24 h and 48 h). (c, d, g) Control.

The probable apoptotic pathway is caused by a rise in ROS levels, which also initiates the pathological alterations such as oxidation of proteins, lipid peroxidation, inflammatory conditions, and destruction of DNA. Similar results from a previous study showing that MgO and silica nanoparticles caused delayed apoptosis among human colon cancer (HT-29) cells were reported [73]. Most anticancer medications enhance apoptosis in cancerous cells by amending the antioxidant and oxidant status levels. As a result, the levels of ROS species in Hep2 cells exposed to CP-MgONPs were estimated by DCFH-DA staining. It was discovered that the green fluorescence intensity was found to be higher in Hep2 cells administrated with CP-MgONPs than in untreated ones, indicating the high content of ROS in treated cells (Figure 8(B)-(g)–(i)), whereas spectrofluorometric analysis was employed to measure the ROS content in CP-MgONPs-administrated Hep2 cells, and the findings revealed that intensity had increased by 68%, displaying that the apoptotic action of CP-MgONPs on Hep2 cancer cells causes a rise in ROS level and its accumulation. In addition, the quantity of antioxidant enzymes such LPO, SOD, GSH, GPx, and catalase as well as oxidant producers like LPO was utilized for assessing the damage produced by oxidative stress in Hep2 cancerous cells after treatment with CP-MgONPs. The results revealed that the MDA levels were 1.2 times higher in CP-MgONPs-treated Hep2 cells in contrast to control group. On the other hand, a decline in the functioning of natural defensive enzyme as well as depletion of nonenzymatic levels of catalase, GPx, SOD, and GSH was observed by 1.2, 2.7, 2.9, and 0.61 times, respectively, in CP-MgONPs-treated Hep2 cells as compared to control group (Figure 9(A)-(a)-(b)). These findings align with a previously disclosed work on ZnONPs-treated HepG2 cells that claimed that alterations in antioxidant/oxidant levels were responsible for the induction of apoptosis [74]. After inducing apoptosis, one of the most essential intracellular processes is the perturbation of mitochondrial membrane potential (MMP). Since mitochondria are the major cell structure responsible for producing ROS, any situation that depletes antioxidants or leads to an overabundance of ROS will activate mitochondrial damage. Loss of MMP, a crucial component of apoptosis, has been caused by the rise in ROS levels [75]. The influence of CP-MgONPs on the MMP loss was assessed by rhodamine 123 staining in this study. Rhodamine 123 with high green fluorescence is a lipophilic cationic dye that exhibits intense green fluorescence may easily pass through undamaged mitochondria and accumulate considerably in their inner membrane. CP-MgONPs-treated Hep2 cells exerted low intensity of green fluorescence of MMP, but high levels of green fluorescence were noticed in untreated cells implying healthy mitochondria (Figure 9(B)-(A)–(C)). Spectrofluorometric estimation revealed a thirty percent drop in fluorescence intensity in Hep2 cells administrated with CP-MgONPs in contrast to untreated cells. A previous study has shown that treatment of PVP-coated AgNPs has promoted ROS production and thereafter MMP depletion in acute myeloid leukemia cells [76]. Furthermore, apoptosis-related nuclear alterations such nuclear fragmentation and condensation were investigated using PI and DAPI staining in Hep2 cells that had been exposed to CP-MgONPs for 24 h and 48 h. The results exhibited condensation/fragmentation of nucleus in CP-MgONPs-treated Hep2 cells after being stained with PI (Figure 9(B)-(d)–(f)) or DAPI (Figure 9(B)-(g)–(i)), suggesting apoptotic changes in nuclei of treated cells, whereas the nuclei of untreated cells were found normal and smooth.

**Figure 10:**
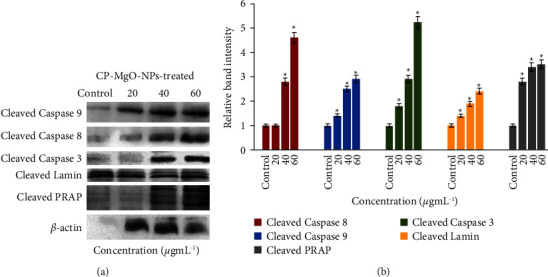
(a) Estimation of cleaved caspase 8, caspase 9, lamin, caspase 3, PARP, and *β*-actin expression in CP-MgONPs-treated Hep2 cells at varied doses for 24 h. (b) Quantification of protein levels in CP-MgONPs-treated Hep2 cells expressed as relative band intensity. Values were presented as mean ± SD (*n* = 3) and showed difference at *P* ≤ 0.05 from the control.

The two principal signaling channels by which apoptosis is normally initiated are intrinsic and extrinsic signaling pathways, which are controlled by caspases 8 and 9, respectively. The extrinsic mediated apoptosis pathway is commenced by interaction between receptors of membrane and external ligand like FAS, followed by binding with adapter proteins related to death motif in the intracellular receptor section. Procaspase 8 is recruited by this death-inducing signaling complex (DISC), which then triggers the stimulation of caspase 8 [77]. Once caspase 8 is activated, it initiates the cascade signaling that eventually leads to cell demise by activating other caspases of execution like caspase 3, while the intrinsic route is activated by an increase in ROS content and eventually causes cytochrome C to be expelled and MMP to fail continuously. The enzymes pro-caspase 9, apoptotic protease activating factor 1 (Apaf-1), and dATP combine with the liberated cytosolic cytochrome C to form an apoptosome complex [78]. More than a hundred receptors, including lamins, PARP, and several DNA-related proteins that detect and activate signals for breaks in DNA strands, are cleaved by active caspase 3 after it has been triggered by the apoptosome complex through a downstream signaling cascade. PARP is broken by the active caspase 3, and broken segment of PARP is one of the indicators of apoptosis [79]. To comprehend the underlying molecular process in CP-MgONPs-treated Hep2 cells inducing apoptosis, the expression of caspase 3, caspase 8, caspase 9, PARP, and lamin was evaluated. It was noticed that Hep2 cells treated with CP-MgONPs exhibited alleviated manifestation of active caspases-3, 8, and 9, PARP, and lamin with the increase in concentration of CP-MgONPs (Figure 10(a)). The densitometry estimation showed fold enhancement in the manifestation of active caspases-3, 8, and 9, PARP, and lamin by 4.1, 1.9, 3.8, 2.5, and 1.1, respectively, after 24 h in CP-MgONPs-administrated Hep2 cells in contrast to control cells at 60 *μ*g·mL^−1^ concentrations (Figure 10(b)). These results demonstrated that CP-MgONP-induced Hep2 cell death involves both intrinsic and extrinsic apoptotic mechanisms. Comparable outcomes were noted in green synthesized silver nanoparticles prepared from *A. calamus* by Nakkala et al. [80].

### 3.4. Photocatalytic Activity

**Figure 11:**
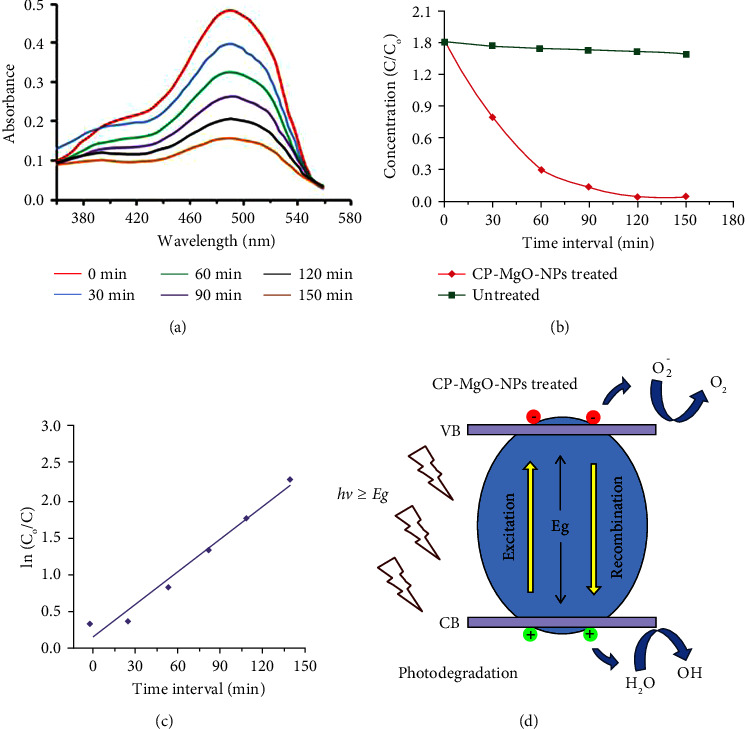
(a) Absorbance spectra of degraded AO-8 dye solution, (b) the C/Co vs time interval graph for the biogenic CP-MgONPs, (c) first-order kinetics graph for the photodegradation of the AO-8 dye, and (d) schematic demonstrating the photocatalytic degradation process of AO-8 dye in presence of biogenic CP-MgONPs.

The photocatalytic effect of biogenic CP-MgONPs produced from citron waste peel extract was tested by degradation of AO-8 dye by photocatalysis under UV light irradiation for 150 min at ambient temperature. After UV light exposure, the absorption peaks' intensity steadily declines over time without altering their positions, and there exists a direct correlation between concentration and dye degradation absorbance.  Figure 11(a) displays the absorbance spectra of CP-MgONPs and the percentage rate of AO-8 dye degradation during time periods ranging from 0 to 150 min. Although the rate of dye degradation percentage initially was seen to be low, it later grew when exposure duration was prolonged as shown by the results. The biosynthesized CP-MgONPs have shown 94% of dye decoloration in 480–490 nm absorbance range, according to the graph plotted between degradation percentages and time interval. The outcomes revealed that after 150 min of treatment, the AO-8 dye decoloration response was validated by a progressive drop in absorption intensities at *λ*_max_ 480–490 nm, which was caused by the photochemical process used under UV light irradiation. The extent of AO-8 dye decoloration was monitored throughout time intervals in both presence and absence of presynthesized CP-MgONPs. It was interesting to note that the UV light barely degraded the AO-8 dye at all, proving that it cannot be degraded by UV light alone. However, the AO-8 dye was almost completely degraded (94%) in the presence of the biogenic CP-MgONPs (Figure 11(b)). The kinetics of AO-8 dye degradation was commonly described using the Langmuir–Hinshelwood model, and the rate constant was calculated using following equation:

(3) $$\begin{array}{c} R=-\frac{dc}{dt}=\frac{kKC}{1+KC}, \end{array}$$

where *R*, *C*, *t*, *K*, and *k* represent the dye discoloration rate (mg/1 min), dye concentration (mg/l), dye illumination time, adsorption coefficient (l/mg), and rate constant (mg/l min). An extremely diluted solution will have a minuscule concentration (C) and formula would be as under:

(4) $$\begin{array}{c} \ln\frac{C_{O}}{C}=kKt\approx k_{\text{app}}t, \end{array}$$

where *K*_app_ represents the apparent rate constant.

The photodegradation process of AO-8 dye represented by 1^st^ order kinetics graph is illustrated in Figure 11(c) and plotting ln (*C*_*o*_/C) against time produced a straight line whose slope matched the 1^st^ order rate constant. The computed regression coefficient (*R*^2^) and apparent rate constant (*k*_app_) for the presynthesized CP-MgONPs were found to be 0.9769 and 0.0274 min^−1^, respectively. The results attained were consistent with the literature that has been published [81]. The decolorization of presynthesized CP-MgONPs was allowed after 150 min of treatment in the photocatalysis of AO-8 dye.  Figure 11(d) illustrates the AO-8 dye discoloration reaction mechanism. Electron-hole (*e*^−^/*h*^+^) pairs are produced when photons of an appropriate wavelength fall on the presynthesized CP-MgONPs [82].

When UV light was applied to the photocatalyst, elevation of electrons (*e*^−^) from the valence band (VB) to the conduction band (CB) would occur and these electrons interact with the photocatalyst surface to produce superoxide ions (O^−2^). The generated superoxide was protonated to HOO• radicals which then interact with *e*^−^ and holes (*h*^+^) in the valence band to produce H_2_O_2_. Simultaneously, HOO• radicals react with free water to create H_2_O and OH^−^ radicals during the process of oxidation. The decolorization mechanism of CP-MgONPs involved the following steps:

(5) MgO+hυ⟶MgOeCB−+hVB+O2+e−⟶O2•−+H+⟶HOO•HOO•+e−+H+⟶H2O2H2O2+eCB−⟶OH∗+OH−

The degradation of dyes was mainly triggered by the fragmentation of the in situ produced H_2_O_2_ into two ^*∗*^OH/OH^−^. On the contrary, the photogenerated photons *h* + vb might be trapped on catalyst surface, exchanging charge with the OH− ions that are there or with the surface that has H2O adsorbed to it to generate OH^*∗*^ active species as illustrated below:

(6) hvb++H2O⟶H++OH∗hvb++OH−⟶OH∗

On the other hand, the production of super oxide free radicals (O_2_, O_2_^•−^, OH−, or HOO^•^) was facilitated by free electrons (*e*^−^) of conduction band. The facile degradation of AO-8 dye was encouraged by the production of extremely reactive oxygen species (O_2_, HOO^•^, O_2_^•−^, and OH^−^). Owing to the very reactive characteristics of reactive oxygen species (ROS), they amalgamated to generate H_2_O_2_ molecule. The H_2_O_2_ molecule disintegrated into free radicals (^•^OH), which in turn degraded the organic contaminants as demonstrated by the equations:

(7) CP−MgONPs h++H2O⟶H++CP−MgONPs OH•CP−MgONPsh++OH•⟶CP−MgONPs OH•2h++2H2O⟶2H++H2O2H2O2⟶2OH•CP−MgONPse−+O2⟶CP−MgONPsO2•−O2•−+2OH•+H+⟶O2+H2O2,H2O2⟶2OH•O2•− or 2OH•+AO−8⟶H2O+CO2

The decrease in photogeneration efficiency may be due to recombination caused by photogenerated *e*^−^/*h*^+^ not being able to reach their proper locations. The structural and morphological features would affect the photocatalytic performance since the photocatalytic activity was enabled across the surface of the MgO nanoparticles during their formation. The highly crystalline and homogeneous MgO nanoparticles may facilitate efficient photodegradation by lowering the recombination rate of photogenerated *e*^−^/*h*^+^ pairs [83]. The efficient AO-8 dye degradation indicates that CP-MgONPs can act as an efficient photocatalyst to break down organic dyes when exposed to UV radiation. The CP-MgONPs' reusability as a photocatalyst in the AO-8 dye decomposition procedure was also investigated. Prior to being used for each cycle, the nanoparticles were carefully rinsed three times in a centrifuge. Three times the photodegradation method has been carried out with comparable circumstances, and degradation outcomes for first, second, and third cycles showed 94%, 83%, and 72% of AO-8 dye degradation, respectively. The results demonstrated that the formed CP-MgONPs have excellent reusability and photostability. Prior study has shown almost similar pattern of photocatalytic potential for gold nanoparticle obtained from orange peel extract [84].

**Scheme 1:**
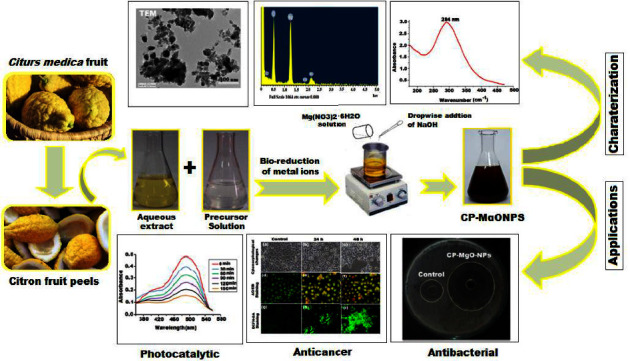
Extraction, synthesis, characterization, and biological application of biogenic CP-MgONPS using waste peels of citron.

Thus, the potential biological applications of formed CP-MgONPs make them an appropriate choice of switching out for nanoparticles made chemically. Additionally, these CP-MgONPs could be used as antimicrobial and anticancer agent, which would offer a potential remedy for the current crisis. However, *in vitro* and *in vivo* research is necessary to learn about their biological characteristics and potential applications. The whole study has been summarized as graphical abstract (Scheme 1). We firmly believe that in the near future, utilization of wastes peels from different citrus plants for the synthesis of nanomaterials will open new way for variety of biomedical applications as nanodrugs [85]. More research is still needed to comprehend and identify the exact molecular process by which plants generate MgONPs so that their size and form may be more effectively regulated. The processes underlying the prolonged toxicity, diffusion, absorption, and excretion of these nanoparticles are currently very little understood.

## 4. Conclusion

In conclusion, this study clearly offers affordable, environmentally benign, and simple reproducible strategy for the synthesis of morphologically distinct CP-MgONPs by employing citron waste peels as reducing, capping, and stabilizing agents. The biosynthesized CP-MgONPs were comprehensively characterized by advanced spectroscopic (UV-Vis, FTIR, and XRD) and modern microscopic (SEM, EDX, and TEM) techniques. The FTIR result identifies numerous phytochemicals involved in the reduction of ions, leading to CP-MgONPs formation. Biosynthesized CP-MgONPs were found to be well-dispersed, relatively stable, and comparatively smaller in size and shape and attached to an organic layer that included flavonoids in the reaction mixture. The SEM monographs showed that average diameter of formed CP-MgONPs was 34.45-52.13 nm range with polyhedral shape. The biosynthesized CP-MgONPs demonstrated antibacterial and anticancer potential in significant levels in a dose-dependent manner. The explored CP-MgONPs exerted potent antibacterial effectiveness towards particular Gram-negative and Gram-positive strains of bacteria. Although the biogenic CP-MgONPs showed toxicity at higher doses, they exerted outstanding efficacy (almost destroyed 95% of cells) against Hep2 cell line, indicating that they might be the potential alternative for killing the cancer cells at optimum doses. Furthermore, the as-prepared CP-MgONPs showed efficient degradation ability against AO-8 dye in the presence of UV irradiation and about 94% of dye was degraded within 150 min. Thus, the CP-MgONPs can be employed as multifaceted therapeutics for diverse biological and biotechnological applications. Their biomedical properties and application can be further investigated using *in vitro* and *in vivo* studies.

## Figures and Tables

**Table 1:** Evaluation of antibiogram resistance profiles of the tested bacterial strains.

Pathogen	AUG	AK	CIP	GEN	NOR	TET	AMP	CFX
*Bacillus cereus*	S	S	S	S	S	S	S	R
*Escherichia coli*	S	S	S	S	S	S	R	R
*Klebsiella pneumoniae*	S	S	S	S	S	S	R	R
*Staphylococcus aureus*	S	S	S	S	S	S	R	R
*Streptococcus pneumoniae*	S	S	S	S	S	S	S	R
*Pseudomonas aeruginosa*	S	S	S	S	S	R	NE	S

S (susceptibility), R (resistance), NE (not evaluated), AUG (Augmentin), AK (amikacin), CIP (ciprofloxacin), GEN (gentamicin), NOR (norfloxacin), TET (tetracycline), AMP (ampicillin), and CFX (cefotaxime).

**Table 2:** The value (mm) of mean zones of inhibition produced after treatment with as-synthesized CP-MgO-NPs using citron (*Citrus medica*) peel extract.

Pathogen	Concentrations (*μ*g·mL^−1^)			
	25	250	500	2500
*Bacillus cereus*	14.21 ± 0.19^c^	14.78 ± 0.27^c^	16.21 ± 0.16^c^	16.78 ± 0.07^b^
*Escherichia coli*	9.72 ± 0.24^a^	15.68 ± 0.13^c^	18.12 ± 0.22^d^	20.72 ± 0.33^c^
*Klebsiella pneumoniae*	7.21 ± 0.08^a^	9.74 ± 0.31^a^	12.24 ± 0.22^a^	14.24 ± 0.09^a^
*Staphylococcus aureus*	12.78 ± 0.21^b^	15.25 ± 0.03^c^	16.78 ± 0.10^c^	19.52 ± 0.05^c^
*Streptococcus pneumoniae*	8.63 ± 0.17^a^	9.45 ± 0.08^a^	12.89 ± 0.01^a^	13.92 ± 0.01^a^
*Pseudomonas aeruginosa*	11.26 ± 0.02^b^	12.38 ± 0.11^b^	14.68 ± 0.12^b^	16.56 ± 0.06^b^
Gentamicin				27.75 ± 0.2

Superscripts (a, b, c, d) indicate the mean ± standard deviation of the bacterial growth inhibition zone diameter data in triplicates for each concentration produced by biogenically synthesized CP-MgO-NPs on the tested bacterial strains. The values with the same alphabetic superscript in each concentration column were not statistically different from each other.

**Table 3:** Minimum inhibitory concentration (MIC) and minimum bactericidal concentration (MBC) of the MgONPs.

Pathogens	MgONPs	
	MIC (*μ*g·mL^−1^)	MBC (*μ*g·mL^−1^)
*Bacillus cereus*	0.64	1.74
*Escherichia coli*	625	1025
*Klebsiella pneumoniae*	0.78	1.96
*Staphylococcus aureus*	625	1012
*Streptococcus pneumoniae*	0.88	2.11
*Pseudomonas aeruginosa*	0.64	1.89

## Data Availability

The results of this investigation corroborate the findings and are presented within the paper.
