# Supplementary material for: Biogenic Synthesis of Photosensitive Magnesium Oxide Nanoparticles Using Citron Waste Peel Extract and Evaluation of Their Antibacterial and Anticarcinogenic Potential
Source: Bioinorg Chem Appl. 2024 Jun 6;2024:8180102. doi: 10.1155/2024/8180102 (PMC11221967; doi:10.1155/2024/8180102)
Supplement: Supplementary Materials — Supplementary data to this article can be found online. [file 8180102.f1.docx]

**Supplementary file (S1)**

**
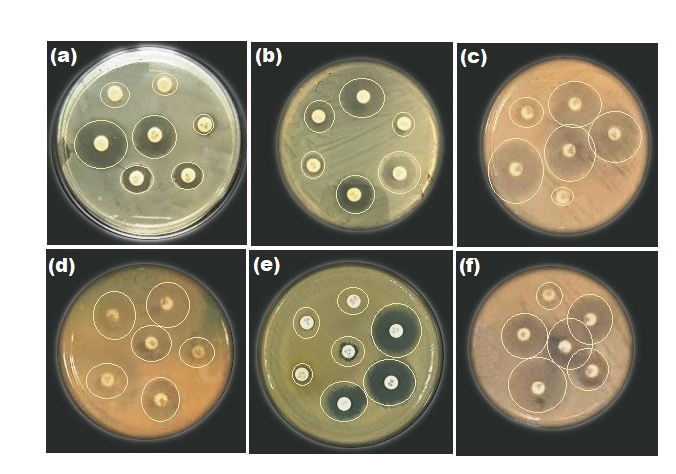
**

**S1.** Zone of inhibition against pathogenic strains (a) *B. cereus,* (b) *E.coli*, (c) *K. pneumonia*, (d) *S. aureus*, (e) *S. pneumonia*, and (f) *P. aeruginosa* in antibiogram tests
